# Environmental Effects on Fractional Exhaled Nitric Oxide in Allergic Children

**DOI:** 10.1155/2012/916926

**Published:** 2011-11-17

**Authors:** Stefania La Grutta, Giuliana Ferrante, Velia Malizia, Fabio Cibella, Giovanni Viegi

**Affiliations:** ^1^Health and Environment Unit, Regional Agency for Environment Protection/ARPA, Sicilia, Corso Calatafimi 217, 90129 Palermo, Italy; ^2^Institute of Biomedicine and Molecular Immunology, “Alberto Monroy,” National Research Council of Italy, Via Ugo La Malfa 153, 90146 Palermo, Italy; ^3^Department of Sciences for Woman and Child's Health, School of Pediatrics, University of Palermo, Via del Vespro 129, 90100 Palermo, Italy; ^4^Institute of Clinical Physiology, National Research Council (CNR) of Italy, Via Trieste 41, 56126 Pisa, Italy

## Abstract

Fractional exhaled nitric oxide (FeNO) is a non-invasive marker of airway inflammation in asthma and respiratory allergy. Environmental factors, especially indoor and outdoor air quality, may play an important role in triggering acute exacerbations of respiratory symptoms. The authors have reviewed the literature reporting effects of outdoor and indoor pollutants on FeNO in children. Although the findings are not consistent, urban and industrial pollution—mainly particles (PM_2.5_ and PM_10_), nitrogen dioxide (NO_2_), and sulfur dioxide (SO_2_)—as well as formaldehyde and electric baseboard heating have been shown to increase FeNO, whilst ozone (O_3_) tends to decrease it. Among children exposed to Environmental Tobacco Smoke (ETS) with a genetic polymorphisms in nitric oxide synthase genes (NOS), a higher nicotine exposure was associated with lower FeNO levels. Finally, although more studies are needed in order to better investigate the effect of gene and environment interactions which may affect the interpretation of FeNO values in the management of children with asthma, clinicians are recommended to consider environmental exposures when taking medical histories for asthma and respiratory allergy. Further research is also needed to assess the effects of remedial interventions aimed at reducing/abating environmental exposures in asthmatic/allergic patients.

## 1. Introduction

Elevated levels of nitric oxide in exhaled air (fractional concentration of exhaled nitric oxide, FeNO) are considered a noninvasive marker of airway inflammation in asthma and respiratory allergy management [1]. Nitric oxide is produced endogenously in the airways from L-arginine by NO synthase. There are two constitutive and one inducible isoforms involved in airway inflammation; their expression is stimulated by inflammatory cytokines [2]. 

FeNO levels correlate with eosinophilic counts in induced sputum or bronchoalveolar lavage fluid and with eosinophil infiltration of the airways, especially of atopic subjects. Indeed, the interest in FeNO is based on the assumptions that FeNO is a useful noninvasive marker of asthma and asthma control [1]. In fact, in mild-to-moderate persistent asthmatic children followed by Childhood Asthma Research and Education (CARE) Network supported by NHLBI, a highly significant correlation of FeNO levels with blood eosinophilia was found [3]. 

FeNO assessment is simple to perform and acceptable for the population, especially in the pediatric age [4]. In addition, FeNO is faster and easier to obtain than other measurements of inflammation such as sputum eosinophils level [5]. For these reasons, some authors use it as a complementary tool to lung function tests in order to obtain a better control of clinical symptoms and asthma exacerbations [6]. 

There is extensive evidence that FeNO is elevated in patients with untreated asthma, whilst it is decreased as a consequence of corticosteroid therapy [2]. Besides, high FeNO levels can suggest a subclinical inflammation of the airways, even in the absence of symptoms and impairment of lung function [6]. Thus, FeNO could represent a helpful tool for rationalizing the anti-inflammatory therapy in patients with respiratory allergy. However, data on long-term benefits of incorporating this kind of measurement in treatment decisions are still missing [1]. 

In clinical practice, increased FeNO levels are used as predictors of failed steroid reduction in stable asthmatic children [7]. FeNO values have been related to the occurrence of disease exacerbation during a 1 yr of followup in moderate asthmatics by Gagliardo et al. [8], but not by Cabral et al. [5]. Such inconsistency has led the authors of a recent systematic review to not include FeNO among the useful clinical predictors of future asthma exacerbations for all the children with moderate-to-severe asthma undergoing ICS tapering [9]. 

In the last decades, there has been an increase in the prevalence of asthma and allergic diseases, particularly among children living in the urban areas of economically developed countries. This has led to suppose that environmental factors, especially indoor and outdoor air quality, may play an important role in the development of allergy and in triggering acute exacerbations of respiratory symptoms [10]. The role of air pollution in the epidemics of allergies is still debated, even if experimental studies have suggested that the effects of air pollutants on the development and the worsening of allergies are biologically plausible. Children are particularly vulnerable because they inhale a higher volume of air per body weight, their lungs are growing, their immune system is incomplete, and their defence mechanisms are still evolving, with respect to adults. If lung defences are breached, normal developmental and homeostatic processes can be disrupted. This could determine disturbances in lung development and acute damage that can, in turn, lead to a chronic reduction in lung function. Therefore, a damage to the developing lung may reduce the maximal attainable functional capacity, reducing the functional reserve in adulthood, thereby, enhancing susceptibility to the effects of ageing, infections as well as pollutants [11]. 

The aim of this review is to make a reappraisal of the current evidences on whether environmental factors, such as outdoor and indoor pollutants, affect FeNO in children. 

## 2. Outdoor Pollutants and FeNO

Since airway inflammation is a hallmark of asthma, FeNO measurement is potentially useful to evaluate the impact of air pollution on the inflammatory state of airways in asthmatic children. Indeed, it is known that air pollution is associated with FeNO in elderly adults with asthma [12], in healthy adults [13], and in schoolchildren [14]. 

There is extensive evidence that outdoor pollutants present in urban areas do have adverse effects on the respiratory health of children [15]. Children in general, mainly those suffering from asthma, are particularly sensitive to the effects of outdoor pollutants such as ozone (O_3_), particulate matter (PM_10_, PM_2.5_), nitrogen dioxide (NO_2_), and sulphur dioxide (SO_2_) [16]. 

Although the mechanisms involved in the bronchial inflammation due to pollution exposure are not yet fully clarified, it is known that the type of air pollutant plays a main role. In this sense, the oxidative stress, induced by reactive oxygen species (ROS), may activate some transcription factors, followed by cytokines secretion and inflammatory cells recruitment. At last, NO is produced by epithelial cells through the induction of inducible NO synthase (iNOS) [17]. Furthermore, experimental evidences suggest that PM organic components have adjuvant effects on airway inflammation, partly through exposure to redox-active chemicals and oxidative stress [18]. PM organic components can also decrease lung function in both elderly adults with COPD and children with asthma [19]. 

 Delfino et al. [2] found that personal (i.e., measured by wearable monitors) and ambient air pollution correlate with increased FeNO concentration from the lower airways of children with asthma. In particular, in two pollutant models, the most robust positive association with FeNO levels was found for personal and ambient elemental carbon and nitrogen dioxide, and for personal but not ambient PM_2.5_. The association between PM and airway inflammation may be missed using ambient particle mass concentration, which may not adequately represent causal pollutant components from fossil fuel combustion. Therefore, the contrasting results for personal versus ambient air pollution could suggest that protecting public health using only a particle mass-based standard may be not sufficient. Supplemental measurements of particle composition and ultrafine particles are needed to better assess the health impact of particulate air pollution. 

Rusconi et al. [20] evaluated lung function and markers of inflammation and oxidative stress in children and adolescents with and without asthma or wheezing symptoms living in a petrochemical polluted area versus those living in a reference area in Sardinia. They found that children living in the polluted area showed decreased lung function (FEV_1_, FEF_25–75_) and increased levels of  FeNO in conjunction with the increased level of certain pollutants, particularly PM_10_ and SO_2_. More recently, Renzetti et al. [10] have found significantly decreased FeNO concentrations after relocating to a rural environment asthmatic children who previously had more active airway inflammation, while living in a highly polluted urban environment.

Similar results were found by Flamant-Hulin et al. [21] who showed significantly increased FeNO levels in both asthmatic and nonasthmatic schoolchildren exposed to high concentrations of formaldehyde, acetaldehyde and PM_2.5_. Stronger associations were found in nonasthmatic children who were atopic, suggesting that they are more sensitive to air pollution than nonatopic children. In other words, atopy and asthma appear as cofactors in determining elevated FeNO levels. In this sense, atopic status is strongly associated with high FeNO levels, even in asymptomatic individuals. The relation between atopy and FeNO levels indicates that FeNO measurements may help to clarify the relevant role of sensitization in the complex interplay of multiple factors determining the translation into clinical allergy. 

Liu et al. [22] demonstrated an important decrement in small airway function and an increase in airway oxidative stress in asthmatic children in association with exposure to SO_2_, NO_2_, and PM_2.5_, but they did not find statistically significant changes in FeNO associated with these pollutants. The authors advance some possible interpretations for their findings: need of a larger sample size to detect significant changes in airway inflammation and measurement of FeNO at low flow rate (0.05 L/sec) to capture inflammation in lower airways. Another explanation might be related to the severe inflammation of the airways that overwhelmed the effects of air pollution, particularly at low concentrations of exposure. FeNO had a statistically significant negative association with O_3_. This result is counterintuitive because laboratory studies showed that high levels of O_3_  cause inflammation in the airways of human subjects. Therefore, a sound interpretation for this negative association remains to be found. Indeed, Kim et al. [23], in an occupational setting, found a significantly inverse relationship between PM_2.5_  exposure and FeNO concentrations, the latter decreasing while PM_2.5_  concentrations increased. 

More recently, Berhane et al. [24] have shown that short-term increases in PM_2.5_, PM_10_, and O_3_ were significantly associated with higher FeNO levels, being PM_10_ effects significantly higher in the warm season. In addition, the effects of PM_2.5_  and PM_10_  had relatively shorter lag structures compared to those of O_3_ that had a longer lag structure (23 days) prior to FeNO measurement. The biologically plausible reasons for the lagged effects of ambient air pollutants on FeNO might depend on the different levels of exposures to pollutants across geographical regions and seasons and on the variable degree of susceptibility of subjects, in terms of asthma and/or allergy status. The authors suggest that current level of ambient pollutants determine a potential increase of nitrosative stress in both healthy and susceptible children, leading to an increase of FeNO.

## 3. Indoor Pollutants and FeNO

Since people generally spend the majority of their time indoors, there is growing scientific evidence that indoor pollution plays a significant role in affecting health. Indoor environment contributes significantly to human exposure to pollutants through complex interrelationships with outdoor pollution [25]. Indoor airborne pollutants are known to trigger allergic responses in asthmatic patients with consequent airway inflammation [26]. Studies in both adults and children showed that sensitization to indoor allergens is associated with an increase in FeNO [27]. In a review by Sofia et al. it is suggested that FeNO can be used as a marker for adverse respiratory health effects caused by indoor air pollution [28]. 

Allergen sensitization may play an important role in elevating NO production in the airways. In a study conducted by Cibella et al. [4], only sensitizations to *Dermatophagoides *and to cat dander were found to influence FeNO levels. This result was confirmed in other studies [29, 30]. Similarly, Leuppi et al. [31] showed that in atopic children an increased FeNO level is associated with sensitization to perennial allergens, possibly through long-lasting inflammatory stimuli, but not with seasonal allergen. Spanier et al. found that cat and dog sensitizations were associated with increased FeNO [29]. Differently, Kovesi and Dales [32] found that dog ownership, but not cat ownership, was associated with changes in FeNO levels. 

Numerous factors related to housing have been associated with airway inflammation in children. Kovesi and Dales reported that, compared with forced air and hot water radiant heat, electric baseboard heating is associated with a higher FeNO [32]. The authors, based on others' report that forced air heating is linked to lower indoor dust mite levels, speculate that the increased levels of indoor dust mite associated with electric heating may increase the likelihood of allergic sensitization and FeNO. In addition, it has been found that electric baseboard heating is related to higher formaldehyde concentrations in houses [33], which, in turn, is associated with increased FeNO levels in children [34]. As far as the exposure to indoor PM sources is concerned, a recent work found that self-reported exposure to the use of woodstoves, candles, or gas cookers was not significantly associated with increased levels of FeNO [35, 36]. 

Pasquale et al. [36] tested the hypothesis that chlorine exposure is associated with increased concentrations of exhaled NO, as a marker of eosinophilic airway inflammation, in children regularly attending (for 1 to 2 hours a week) swimming pools. FeNO level was similar in children who regularly attended a swimming pool and in those who did not, whereas it was higher both in children with upper airway infections in the last week and in those who had a history of asthmatic symptoms. This suggests that intermittent exposure to chlorine derivatives does not induce eosinophilic airway inflammation. 

In addition, two studies evaluated the influence on FeNO of exposure to polyvinyl chloride (PVC) material which today represents a common indoor pollutant. Tuomainen et al. [37] did not observe changes in FeNO levels in exposed individuals. On the contrary, Kolarik et al. [38] found a significant increase of FeNO compared to the reference condition (clean outdoor air), suggesting that exposure to plastic materials can be associated with a subclinical inflammation of the airways.

## 4. Smoking and FeNO

Many studies investigated the effects of smoking on FeNO values in both adults and children. There is consistent evidence that active smoking and acute cigarette smoke exposure lead to a transient decrease in FeNO levels in healthy and asthmatic adults [39, 40]. As far as we know, no study has yet demonstrated a link between passive smoke and FeNO values in healthy children. In asthmatic children, results are discordant probably due to methodological biases (small sample sizes, heterogeneous study populations, lack of control for potential confounding factors) [41]. Different studies have not found a significant association of FeNO and environmental tobacco smoke (ETS) exposure in children with asthma [32, 42]. In particular, Laoudi et al. [41] observed lower FeNO levels in exposed asthmatic children than in unexposed children. This could be explained by different mechanisms according to the type of exposure. Acute exposure induces a marked but transient reduction in FeNO levels related to a negative feedback of iNOS activity, since tobacco smoke contains high concentrations of NO. In the case of daily exposure, the mechanism is still unknown, but one plausible hypothesis is that the progressive negative feedback leads to the inhibition of iNOS gene expression. 

Genetic differences may explain some of the conflicting results in studies evaluating the effects of tobacco exposure on FeNO levels. Spanier et al. found that a NO synthase gene (NOS3) polymorphism (a mutation in exon 7) modifies the effect of nicotine exposure on FeNO. The authors noticed that this polymorphism determines decreased FeNO levels in children exposed to increasing nicotine concentrations, possibly through a decreased enzyme activity due to a combination of genetic and environmental factors [43]. 

More recently, Salam et al. [45] have shown that common variants in the NO synthesis pathway genes contribute to variation in FeNO levels in children. Particularly, the authors found that four NOS2A single nucleotide polymorphisms (SNPs) and one ARG2 SNP are significantly associated with lower FeNO. They also noticed that the ARG2 SNP modify the effect of NOS2A on FeNO. Therefore, FeNO levels depend on variants in both ARG2 and NOS2A. This gene-gene interaction may be due to a competition for a common substrate, L-arginine, since arginase can inhibit iNOS expression reducing NO synthesis. Some of the observed genetic influences were stronger in children with asthma. Therefore, asthma status can be considered an important factor for determining the contributions of these genetic variants to FeNO levels. 

## 5. Variations and Inconsistencies in FeNO Measurements in Children Exposed to Environmental Pollutants

Most of referred data show that FeNO assessment is a complementary tool to evaluate the effects of environmental pollutants exposure in children. Nevertheless, some variations exist among the studies. Such variations are mainly related to the population studied (i.e., genetic variation, atopic versus nonatopic, and asthmatic versus nonasthmatic), including the treatment effects (i.e., inhaled steroid in asthmatics), and to the variable pollutants exposure (i.e., personal versus ambient, level of exposure, and short term versus long term). 

Up to date, there are only few data about the influences of genetic variations of NO synthesis pathway on FeNO levels, suggesting that the genetic factors play a key role in determining FeNO levels and have to be considered to understand interindividual differences, especially when there are host susceptibility factors, such as asthma and/or atopy.

Previous studies found that atopy status is *per se* able to significantly influence FeNO levels, even in asymptomatic individuals [21, 31]. In our experience, a significant relation exists between FeNO levels and number of positive skin tests, and the highest FeNO levels are observed in atopic children with physician-diagnosed asthma. Thus, the association of asthma and atopy appears to be the most consistent predictor of increased FeNO level [4].

Earlier studies showed that FeNO levels are raised in asthmatic children, especially if asthma is uncontrolled and during asthma exacerbation. Instead, FeNO levels are reduced after corticosteroid treatment [1, 44]. Therefore, asthma condition may be an effect modifier in the relationship air pollution FeNO; it is associated with high FeNO levels according to several authors [2, 10, 45], with decreased FeNO according to others [22, 43]. 

The contrasting results of FeNO values for ambient and personal air pollution may be related to the individual susceptibility and to the considered pollutant. In this sense, Berhane et al. [24] underline that current levels of ambient pollution have the potential to increase nitrosative stress in both healthy and susceptible children. Heterogeneity is increased by the different level of exposure reported in the studies; anyway, it allows investigating a dose-response effect, mainly at the proximity level of individuals [2]. Indeed, the use of multipollutant models might improve in the final interpretation of interaction of pollution exposure with FeNO values. 

In agreement with Berhane et al. [24], we think that the inconsistencies on FeNO level interpretation about the duration of the lags might be overcome including the time-activity patterns to avoid the misclassification of exposure assignments.

 Finally, in light of the evidence that the variations in FeNO measurements show many inconsistencies in children exposed to environmental pollutants, further research is warranted to examine whether FeNO could be used as a useful tool to identify the most susceptible children to adverse respiratory effects from exposure to pollutants.

## 6. Conclusion

Since many factors such as atopy, sex, season, and corticosteroid treatment influence FeNO values [4, 29], clinicians and researchers should know an individual FeNO baseline in asthmatic children management before studying the effect of other determinants. In addition, clinicians should take into account some indoor pollution factors, like indoor allergens [4, 31, 32], mainly Dermatophagoides and pets, electric baseboard heating [32], higher formaldehyde concentrations in houses [34], using of woodstoves, candles or gas cookers [35, 36], chlorine, or PVC exposure [36, 38] for which increase FeNO values are found in children. Furthermore, the possibility that a low FeNO level in an asthmatic child is related to ETS exposure should be considered by the clinicians [41–43, 45]. 

As environmental interventions are an important component of asthma management, FeNO might be useful to integrate the control of environmental triggers into asthma management [29]. Assuming that the inflammatory response of the airways to airborne irritants is reversible, it may be expected that limiting air pollution will reduce airway inflammation [10]. Therefore, FeNO assessment may help in the management of asthmatic patients when they are exposed to major changes in environmental disease-related factors [46, 47]. 

Even considering that we reviewed this topic from a public health perspective, the literature impacts into the clinical practice should include the more recent findings on the higher FeNO levels significantly associated with the short-term increases in PM_2.5_, PM_10_, and O_3_. Therefore, we would recommend clinicians to obtain more complete information on outdoor and indoor exposure of susceptible subjects for the sake of an accurate interpretation of the FeNO levels in the real life management of allergic asthmatic children. The observed reduction of the FeNO levels in asthmatic children after one week relocation to the rural environment [10], the lower level of FeNO in children living in the reference area in comparison to those living in the high polluted area [20], as well as the higher FeNO levels in children living in homes with high average of formaldehyde levels versus those living at a lower concentration [34] suggest that FeNO assessment might be a useful marker also to monitor the variation in airway inflammation due to the pollution exposure. 

In light of the reviewed evidence, we would recommend that further research is carried out firstly by organizing a cross-sectional multicentre epidemiological study in different countries characterized by different genetic background and level of environmental pollutants in order to better investigate the effect of gene and environment interactions on FeNO levels; such study design would also help obtaining reliable reference values of FeNO. Subsequently, nested case-control studies should be performed in order to assess the impact of remedial interventions regarding the previously cited factors affecting a FeNO concentration; such study design would allow clinicians to implement a public health perspective in the individual-physician relationship. Lastly, it would be helpful to expand the medical histories of children enrolled in pharmaceutical clinical trials, through collecting information on exposome, in order to reduce the residual variability of therapeutic treatment.
